# Research on Named Entity Recognition Based on Multi-Task Learning and Biaffine Mechanism

**DOI:** 10.1155/2022/2687615

**Published:** 2022-08-25

**Authors:** Wenchao Gao, Yu Li, Xiaole Guan, Shiyu Chen, Shanshan Zhao

**Affiliations:** School of Mechanical Electronic & Information Engineering, China University of Mining & Technology, Beijing 100083, China

## Abstract

Commonly used nested entity recognition methods are span-based entity recognition methods, which focus on learning the head and tail representations of entities. This method lacks obvious boundary supervision, which leads to the failure of the correct candidate entities to be predicted, resulting in the problem of high precision and low recall. To solve the above problems, this paper proposes a named entity recognition method based on multi-task learning and biaffine mechanism, introduces the idea of multi-task learning, and divides the task into two subtasks, entity span classification and boundary detection. The entity span classification task uses biaffine mechanism to score the resulting spans and select the most likely entity class. The boundary detection task mainly solves the problem of low recall caused by the lack of boundary supervision in span classification. It captures the relationship between adjacent words in the input text according to the context, indicates the boundary range of entities, and enhances the span representation through additional boundary supervision. The experimental results show that the named entity recognition method based on multi-task learning and biaffine mechanism can improve the F1 value by up to 7.05%, 12.63%, and 14.68% on the GENIA, ACE2004, and ACE2005 nested datasets compared with other methods, which verifies that this method has better performance on the nested entity recognition task.

## 1. Introduction

**Figure 1:**
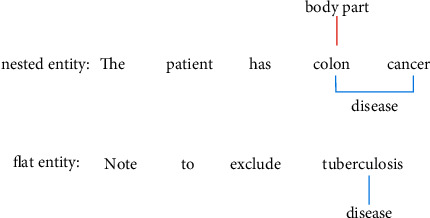
Named entity example.

Named entity recognition tasks are mainly studied for flat entities and nested entities. In the process of many named entity recognition tasks (e.g., GENIA [ref], ACE2004 [ref], and ACE2005 [ref]), many entities may be nested, that is, there are one or more other entities inside an entity. As shown in Figure 1, the sentence “Note to exclude *tuberculosis*” contains only the flat entity “*tuberculosis*”, while the entity “colon cancer” in the sentence “The patient has colon cancer” also includes the entity “colon” to form a nested form. For named entities with nested structure, due to their complex hierarchical structure, the traditional named entity model based on sequence labeling is difficult to deal with directly and effectively. Therefore, increasingly researchers began to pay attention to the problem of nested named entity recognition and proposed some models especially suitable for the task of nested named entity recognition.

Sequence-based methods utilize traditional sequence labeling methods to learn nested structures. Ju et al. [1] proposed a stacked LSTM-CRF model to predict nested named entities by dynamically stacking flat NER layers to identify nested entities. Katuyar and Cardie [2] used their proposed recurrent neural network-based method to handle nested named entity recognition. Lu and Roth [3] introduced a hypergraph structure for learning nested named entities. Wang and Lu [4] further proposed a neural segmentation hypergraph to address the problem of nested entity recognition. Span-based methods are another advanced method for unified named entity recognition. The idea of this class of methods is to enumerate all possible spans and classify them. The span model of Li et al. [5] introduces a general framework by several information extraction tasks that share span representations using dynamically constructed span graphs. Sohrab and Miwa [6] enumerate all possible regions of a latent entity or span, and classify them with deep neural networks. Yu et al. [7] proposed the idea of graph-based dependency parsing to provide the model with a global view of the input through biaffine mechanism, scoring pairs of start and end tokens in a sentence. Use this tag to explore all spans so that the model can accurately predict named entities.

However, these research methods have different problems. The traditional sequence labeling method identifies nested entities layer by layer. And the error of the inner entity recognition will directly lead to the wrong identification of the outer entity, which will lead to the problem of error propagation of nested entities. When the input sentence is too long or there are many entity categories in the hypergraph-based model, the hypergraph structure will become complex, resulting in difficulty in parameter optimization. The span-based method first identifies the head and tail spans of entities, constructs head-tail entity pairs, and then performs label classification. Construct head-tail pairs based on real labels during training, and predict which words are head-tail pairs during testing. This method is easy to detect nested entities in different subsequences. However, due to the emphasis on learning head and tail representations, the model lacks obvious boundary supervision for entities and lacks effective use of entity boundary information. And more entity words are not predicted, which makes the model have the problem of high precision and low recall, thus affecting the overall recognition effect. In addition, when the entity span is too long, the interactive information between the head and tail spans of the entity will gradually decay. And the problem of information interaction between the head and tail spans is also ignored to a certain extent, which affects the recognition effect.

Therefore, in view of the various problems raised above, this paper proposes a named entity recognition model based on multi-task learning and double affine mechanism:

1. To enhance boundary supervision, in addition to using the biaffine model to classify the learned head and tail spans, the model adds an additional boundary detection task to predict words as entity boundaries.
2. The model captures the connection between adjacent words according to the context, trains the two tasks jointly under the framework of multi-task learning, and enhances the span representation through additional boundary supervision.
3. The boundary detection module helps to generate high-quality span representations, more entity words are correctly predicted, and the recall rate of the model is improved, thereby improving the overall effect of the model.

## 2. Materials and Methods

**Figure 2:**
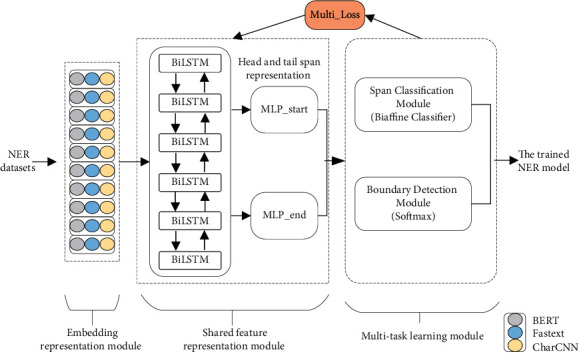
MTL-BAM model structure.

In this work, we propose a model using Multi-Task Learning and Biaffine Mechanism (MTL-BAM). In this model, through multi-task learning, a multi-task loss is applied to simultaneously train two parts, the boundary detection module and the entity span classification module. The MTL-BAM model consists of an embedded representation module, a shared feature representation module, and a multi-task learning module. The specific model structure is shown in Figure 2.

The input of the model is a sentence, and the output is the entity in the sentence and the category corresponding to the entity.

Next, the research content and implementation process are described in three parts: the design of the embedding representation module, the shared feature representation module, and the multi-task learning module.

### 2.1. Embedding Representation Module

In the embedding representation module, in order to obtain the features of the input text more comprehensively, three embedding methods of BERT, CharCNN, and FastText are used. The BERT [8] method can obtain the contextual features of the sentence; the CharCNN [9] method can obtain the character-level text features; the FastText [10] method can obtain the word-level features of the sentence. Next, the three embedding methods are described in detail.

#### 2.1.1. BERT Embedding

BERT passes the words in each input sentence through the word embedding layer and converts them into vector representations. To keep the size of the vector dimension the same, padding operations are performed on training texts of different lengths before word embedding, and the length of each training text is become the same by learning from each other's strengths. In addition to word embedding, the input of BERT also contains two embedding layers: one is sentence embedding, which is used to distinguish whether the current word belongs to sentence *A* or sentence *B*; the other is position information embedding, which obtains the relative position information of the sentence context and expresses the sequence order in which each word appears in the sentence.

**Figure 3:**
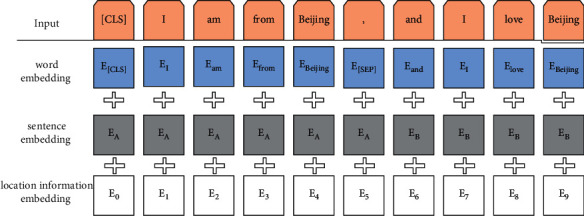
The input representation structure of the BERT.

The input of BERT consists of the summation of the above three embedding vectors, and the structure is shown in Figure 3. Setting [CLS] and [SEP] flags for each input text, [CLS] is used to mark the beginning of a sentence and [SEP] is used to distinguish two sentences. After the above vector representation is obtained, it is input to the bidirectional encoder Transformer to realize the feature extraction of the text sequence. The BERT model is pretrained on a large number of public corpora to form a text vector representation that carries effective information. *X*_*t*_^*lm*^ represents the contextual embedding vector representation of the pretrained sentence at time *t*.

#### 2.1.2. CharCNN Character Embedding

The character embedding layer uses the CharCNN network encoding to map words into character-level vector representations. The specific method is: constructing the input text into a character encoding, sending it to a one-dimensional CNN model, and outputting it with a specific width after one-dimensional convolution. Perform max pooling to obtain character vector representations with specific dimensions. After the processing of the CharCNN network, the character-level vector representation X_*X*_^char^ is obtained, which represents the character embedding vector of the sentence at time *t*.

#### 2.1.3. FastText Word Embedding

The word vector model can map the sentence into a word-level vector representation. This paper uses the word vector pretrained by FastText to obtain the word representation of the sentence, where X_*t*_^word^ represents the word vector representation of the sentence at time *t*.

After obtaining the character vector, word vector, and context representation, the mapped results are spliced and sent to the next network. For a sentence consisting of *t* tokens, output a sequence vector, the vector is shown in the following formula:

(1) $$\begin{array}{c} {\text{X}}_{\text{t}}=\left[{\text{X}}_{t}^{\text{lm}}\;;{\text{X}}_{t}^{\text{char}}\;;{\text{X}}_{t}^{\text{word}}\right]. \end{array}$$

Among them, *t* represents the current time. [; ] represents the connection, and *X*_*t*_^*lm*^， X_*t*_^char^， X_*t*_^word^ , respectively, represent the embedding vector at time *t*. *X*_*t*_ represents the output vector at the current time *t*.

### 2.2. Shared Feature Representation Module

#### 2.2.1. BiLSTM [11]

The shared feature representation module first obtains BERT and other output vectors through the BiLSTM layer, to obtain more comprehensive semantic information. LSTM can effectively solve the phenomenon of vanishing gradient or exploding gradient in recurrent neural networks [12]. BiLSTM is composed of forward LSTM and backward LSTM. The LSTMs in the two directions are connected in series to obtain bidirectional word vector information. This paper adopts the BiLSTM structure to model contextual information. BiLSTM simulates the context-time interaction of sentences after obtaining the embedding vector of the embedding layer. For each sentence, the left-to-right and right-to-left order representations are computed separately.

#### 2.2.2. Head and Tail Span Representation

The head and tail span table module is based on the output of the BiLSTM layer. Use two MLPs on each hidden layer before going to the next layer, creating two different representations (s, e) as the start and end of the entity span. S carries information that identifies the head of the entity, *e* carries information that identifies the tail of the entity, and other redundant information is removed. The two MLP layers learn the head and tail representations of the span and the MLP layer is set to a lower dimension, so as to alleviate the overfitting phenomenon generated by the output of the BiLSTM network and obtain more features in the text.

The encoded representation is input into the classifiers of MLP and softmax, which detects whether the word is the beginning or the end of an entity, and generates a span representation carrying entity head and tail information, respectively. *s*(*t*) and *e*(*t*) are the head and tail span representation of the entity, respectively. Their expressions are shown in formula (2) and (3), where h_t_ represents the hidden layer output of BiLSTM, and MLP_s_ and MLP_e_ represent two multilayer perceptrons processing head and tail information, respectively. For (*s*(*t*), *e*(*t*)) such token pairs, feed each such token pair to the underlying network for the associated task.

(2) $$\begin{array}{c} \text{s}\left(\text{t}\right)=\text{softmax}\left({\text{MLP}}_{\text{s}}\left({\text{h}}_{\text{t}}\right)\right), \end{array}$$

(3) $$\begin{array}{c} e\left(\text{t}\right)=\text{softmax}\left({\text{MLP}}_{\text{e}}\left({\text{h}}_{\text{t}}\right)\right). \end{array}$$

### 2.3. Multi-Task Learning Module

There are two subtasks in the MTL-BAM model: entity boundary detection module and entity span classification module. The two tasks are described as follows.

#### 2.3.1. Entity Span Classification Task

The model used in the entity span classification task is a biaffine mechanism. The biaffine mechanism is different from the traditional MLP mechanism, using a biaffine attention mechanism instead of bilinear, using a bilinear layer instead of two linear layers and one nonlinear layer, simpler than the traditional MLP networks. After obtaining (s, e) above, input the biaffine network to obtain a score matrix.

**Figure 4:**
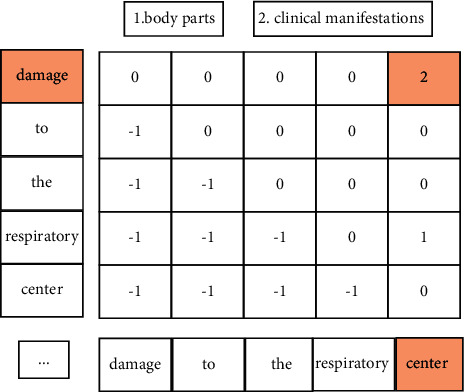
Score matrix for entity span.

Figure 4 shows the entity span matrix constructed for the entity span classification task. In “damage to the respiratory center”, “damage” is the beginning of the entity, and “center” is the end of the entity. The constituted entity goes through the following formula to calculate all scores of the entity type contained in the current data set, and the entity type with the highest category score is clinical manifestation, then “damage to the respiratory center” is identified as the clinical manifestation entity type. This is a fixed-category classification problem, and the prior probability of the head and tail spans needs to be considered at the same time. It is known that words such as head and tail are the posterior probability of a certain category relationship, as shown in the following formula:

(4) $$\begin{array}{c} r_{m}\left(i\right)=s{\left(i\right)}^{T}U^{\left(1\right)}e\left(i\right)+U^{\left(2\right)}\left(s\left(i\right)⊕e\left(i\right)\right)+b. \end{array}$$

For entity fragment *i*, *r*_*m*_ provides the score that the current entity fragment can constitute a named entity category, in the case of restricting the entity's start position before the end position. Among them *s*(*i*), *e*(*i*) represents the head and tail representation of the *i* segment, and ⊕ represents the concatenation of vectors. *s*(*i*)^*T*^ represents the transpose of the *s*(*i*) vector. *U*^(1)^ indicating the posterior probability of the current word being the head and tail entity category at the same time, *U*^(2)^ indicating the posterior probability of the current word being the head or tail entity category, *b* represents the prior probability of not knowing what entity class it is. To determine the entity category spanning the head and tail of an entity, *r*_*m*_(*i*) represents the category score of all possible fragments that currently constitute the named entity. Then, get the category with the highest score as the category predicted for each span, as shown in the following formula:

(5) $$\begin{array}{c} y{}^{\prime }\left(i\right)=\text{argmax }r_{m}\left(i\right). \end{array}$$

After predicting the category of the entity segment, the span of all entity categories is arranged in descending order, and the postprocessing protocol is adopted: for nested entities, it is judged whether there is partial overlap between different entities, and if there is partial overlap, the entity with the highest score is retained. For the *i*th and *j*th entities, *s*_*i*_ and *s*_*j*_, respectively, represent the starting position of the entity, *e*_*i*_ and *e*_*j*_, respectively, represent the end position of the entity. If the partial overlap satisfies the case of *s*_*i*_ < *s*_*j*_ < *e*_*i*_ < *e*_*j*_, the highest-scoring entity and its category are reserved.

The learning objective of entity span classification is to assign a correct class (including nonentities) to each valid interval. Therefore, it is a multiclass classification problem, and the model is optimized with Softmax cross-entropy. The loss of the model is shown in the following formulas:

(6) $$\begin{array}{c} p_{m}\left(i_{c}\right)=\frac{\text{exp}\left(r_{m}\left(i_{c}\right)\right)}{\sum_{\hat{c}}^{C}\text{exp}\left(r_{m}\left(i_{\hat{c}}\right)\right)}, \end{array}$$

(7) $$\begin{array}{c} {\text{loss}}_{b}=-\sum_{t=1}^{N}\sum_{c=1}^{C}y_{ic}\text{log}p_{m}\left(i_{c}\right). \end{array}$$

Among them, *N* represents the length of the sentence, *C* represents the number of entity label types, *y* represents the actual label type of the current word. *y*_*ic*_ is 1 if the current category is *c*, 0 otherwise. *p*_*m*_(*i*_*c*_) is the output of the neural network, that is, the probability that the category is *c*. This output value is calculated using the Softmax mentioned above. Finally, the loss loss_*b*_ of the span classification module is obtained.

#### 2.3.2. Entity Boundary Detection Task

**Figure 5:**
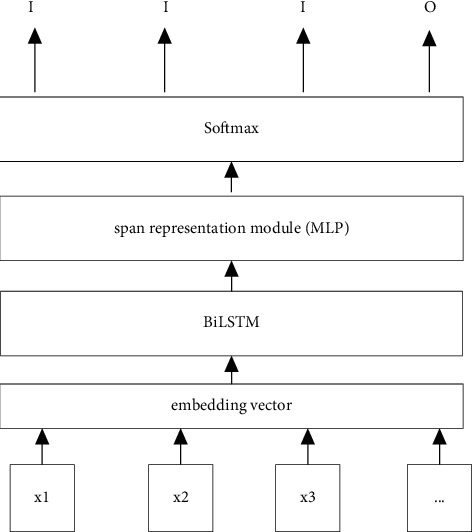
Boundary detection model.

When using the biaffine mechanism for entity span classification, the introduction of head and tail span information can easily identify nested entities. However, the span learning of head and tail makes the model lack clear boundary supervision, which reduces the number of accurate candidate entities learned and reduces the effect of entity recognition. Even for nested entities, the recognition of head and tail spans does not make the connection between internal and external entities, and the lack of accurate external boundary information will cause internal entity recognition errors and reduce the recognition effect. Therefore, a multi-task learning method is introduced, and a boundary detection module is constructed to assist entity category prediction. The boundary detection model is shown in Figure 5.

After obtaining the head and tail span representation, the shared feature representation of multi-task learning is used as input, and the ReLU activation function and Softmax classifier are input to predict boundary labels, and the training speed is faster because Softmax also incorporates the mutual exclusion information between classes. The calculation process is shown in the following formulas:

(8) $$\begin{array}{c} O\left(t\right)=U\left(s,e\right)+b, \end{array}$$

(9) $$\begin{array}{c} d\left(t\right)=\text{softmax}\left(\text{O}\left(t\right)\right). \end{array}$$

For each token in the sentence, here *U* and *b* are trainable parameters. *s* and *e* represent the span representation of header information and tail information, and “,” represents the concatenation operation of vectors. *d*(*t*) is the calculation result of the Softmax network layer, indicating the probability that the current is “O” and “I”. We compute the loss formula (10) between the true distribution $\hat{d}\left(t\right)$ and the predicted distribution *d*(*t*):

(10) $$\begin{array}{c} {\text{loss}}_{d}=-\sum \left(\hat{d}\left(t\right)\right)\text{log}\left(d\left(t\right)\right). \end{array}$$

Since the model shares the same entity boundary when performing entity boundary detection and entity class judgment, the losses for the two tasks of entity boundary detection and entity span classification are jointly trained. In the training phase, the real entity boundary labels of the data are input into the model to train the entity boundary detection classifier to avoid the classifier being affected by false boundary detection during training. During the testing phase, the output of the boundary detection classifier is used to indicate which entity fragments should be considered when predicting the classification labels.

#### 2.3.3. Multi-Task Learning Loss

Therefore, the total loss of the model is the sum of the losses of entity boundary detection and entity category judgment. Total loss is defined as the following formula:

(11) $$\begin{array}{c} \text{Multi}\_\text{Loss}={\text{loss}}_{b}+\alpha {\text{loss}}_{d}, \end{array}$$

*α* is a hyperparameter that is used as a mixing ratio parameter to control the introduction of information to control the importance of the two losses between entity boundary detection and entity category judgment.

Use the boundary detection module to obtain the boundary information of the entity to obtain the internal and context information of the current entity, learn richer features through multi-task learning, optimize the head and tail span representation through the back-propagation of the loss function, and improve the classification of the entity span more accurately. The acquisition of the external information of the entity improves the recognition effect of the inner entity, and the inner entity information will also be transferred to the boundary detection model through the multi-task model to promote the boundary detection effect.

By implementing the above process, the algorithm flow of this model is shown in Table 1.

## 3. Results and Discussion

### 3.1. Experimental Environment Parameter Settings

This paper uses the Windows system for experiments, based on the Python platform, using pycharm as a development tool. The model is constructed using the open source deep learning framework Tensorflow, which is developed and maintained by Google's artificial intelligence team, Google Brain. It is deployed on various servers, PC terminals, and web pages, and supports GPU high-performance numerical computing.

The named entity recognition model (MTL-BAM) based on multi-task learning and biaffine mechanism is experimented on two flat entity datasets JNLPBA [13], CoNLL2003, and three nested entity datasets GENIA [14], ACE2005, and ACE2004 [15]. It is compared with BAM that only uses a biaffine mechanism for entity recognition without using a multi-task learning framework, and the experimental results are compared with other models that achieve some results in the named entity recognition tasks. The evaluation indicators used are the precision rate P, the recall rate *R*, and the F value.

The experimental parameter settings in this paper are shown in Table 2.

The convolution kernel window size of the convolutional layer of the CharCNN network is set to [3–5], and the character vector dimension is 50 dimensions. Use the public pretrained word vector FastText as the choice of word vector, and the word vector dimension is set to 300 dimensions. BiLSTM sets the output dimension of 3 layers to 200 dimensions, and the dimension of two fully connected layers is set to 150 dimensions, reducing the output dimension of LSTM to prevent overfitting. At the same time, to prevent overfitting, the BiLSTM layer and the MLP layer, respectively, set the dropout [16] to 0.4 and 0.2. The Adam optimizer [17] is used as the optimizer for model parameter update during training, and the learning rate is set to 0.001.

### 3.2. Analysis of the Experimental Results of Nested Named Entity Recognition

#### 3.2.1. Analysis of the Experimental Results of the GENIA Dataset

**Figure 6:**
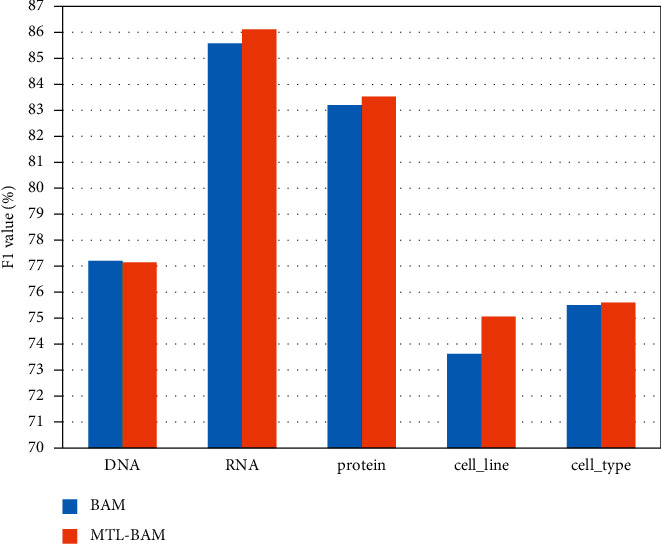
Experimental results of various entity types in GENIA dataset.

The experiments compare the performance of the MTL-BAM model and the BAM model on five different entity categories in the dataset GENIA. The experimental results on this dataset are shown in Table 3. The best results in each group of experiments are shown in bold.  Figure 6 is a histogram of the data distribution corresponding to Table 3.

It can be seen from the figure and table that the recognition result of the RNA entity type is the highest, because the RNA boundary in the data set is generally represented by mRNA and RNA, so the model has learned the boundary information of RNA, and the recognition effect is the best. Therefore, it can be obtained that the entity boundary information plays an important role in the accurate identification of the entity. Except for the 0.05% drop in DNA type data, the *F*1 value of all other entity types has a certain effect improvement, among which the cell_type entity type with the least improvement has increased by 0.06%, and the highest cell_line entity type has increased by 1.44%, the overall *F*1 value increased by 0.22%, indicating that the MTL-BAM model has a certain effect compared with the BAM model. From the bold display in the table, after adding the multi-task learning model, the overall recall rate has been improved compared with before. The reason is that the boundary detection module enhances entity boundary supervision and obtains entity context representation information by obtaining entity boundary information. The span classification module provides a boundary representation, which enables the model to extract more correct entity segments. At the same time, from the perspective of accuracy, the results of the least number of RNA and cell_line have improved, indicating that the boundary detection module strengthens the connection between the inside and outside of the nested entity, so that the sparse entity learns more internal and external features, and the accuracy rate for other entity types is improved. There is a downward trend, and the reason may be that the extracted entity fragments are not accurately classified. The model should also set more effective methods of multi-task learning to strengthen the connection between internal and external entities and improve the accuracy. It shows that the multi-task model can improve the effect of the single-task biaffine mechanism.

#### 3.2.2. Analysis of Experimental Results of ACE2004 and ACE2005 Datasets

In the experiment, the performance of the MTL-BAM model on seven different entity categories of the two nested datasets ACE2004 and ACE2005 was further verified. The experimental results on the ACE2004 and ACE2005 datasets are shown in Tables 4 and 5.

It can be seen from the table that the *F*1 value of the MTL-BAM model on the ACE2005 dataset is 0.39% higher than that of the BAM model, and the recall rate of the other six entity labels is the same except for the FAC entity category. The overall recall rate has also improved by 0.79%. On the ACE2004 dataset, the overall *F*1 value of the model has increased by 0.33%. Except for the same recall rate on the FAC entity category, other recall rates have been improved, and the overall recall rate has increased by 2.08%. The recall rate of the model in this chapter is higher than that of the BAM model on both data, and the overall *F*1 value is also higher than that of the BAM model, which proves the effectiveness of the MTL-BAM model on multitype nested datasets.

#### 3.2.3. Analysis of Nested Data Set Comparison Experiment Results

The MTL-BAM model is compared with some existing neural network-based nested named entity recognition models. The experimental comparison results are shown in Table 6. The precision and recall of the experimental results are shown, and the final comparison with other entity recognition models only uses the *F*1 result as a comparison.

In the table, Ju et al. [1] and Zheng et al. [18] are methods based on sequence annotation, Katuyar, Wang and Lu are methods based on hypergraph, Luan, and Sohrab are span-based method methods. Jana et al. [19] is an approach by using a linear model for nested label encoding. The method proposed in this chapter is higher than all above methods in recall rate and *F*1 value, and the accuracy rate is lower than that of Sohrab in the GENIA dataset, but because the precision rate and recall rate of this model are too different, it shows that the span-based model has a high precision and low recall rate due to incomplete span detection. Compared with Sohrab, the recall rate of this paper is improved by 16.68%, and the overall result is 3.64% higher. For the ACE2004 dataset, the *F*1 value is the highest result of the current model, which is 0.63% higher than the Luan model. The *F*1 value of the model in this chapter is the highest result on the ACE2005 dataset, and the *F*1 value of the model in this chapter is 0.88% higher than that of Strakova. Based on the above conclusions, the model in this paper verifies the effectiveness of recognition in nested datasets by comparing with other models. It is verified that the proposed multi-task framework enables the boundary detection module to enhance the entity boundary supervision and obtain entity context representation information by obtaining entity boundary information, and provides boundary representation for entity span classification module, which improves the effect of entity recognition. At the same time, it shows that the model in this chapter has an important contribution to improving the recall rate and balancing the *F*1 value.

### 3.3. Analysis of Experimental Results of Flat Named Entity Recognition

#### 3.3.1. Analysis of the Experimental Results of the JNLPBA Dataset

The experimentally compared models for the JNLPBA dataset include the models of Wang et al. [20] and Song [21]. The former proposes to share character- and word-level information between related biomedical entities across different labeled corpora. The latter uses BioBERT, a domain-specific language representation model pretrained on a large-scale biomedical corpus, with the same principles as the BERT model. Moreover, it includes some of the models mentioned above.

The comparison results of the MTL-BAM and BAM models on the dataset JNLPBA based on the same experimental environment are shown in Table 7.

On the JNLPBA dataset, the *F*1 value of the model in this paper has increased by 0.35%, the recall rate has increased by 1.25%, and the accuracy has decreased to a certain extent. The experimental results verify that the model is equally effective on flat entities and nested entities.

Table 8 shows the experimental comparison results between the MTL-BAM model and the other entity recognition models mentioned above. Since most models only show the final evaluation index *F*1 value in the JNLPBA dataset, the MTL-BAM model will only compare the *F*1 value.

Compared with the other entity recognition models, it is 1.15% higher than BioBERT using biomedical corpus as a pretraining model, and the verification model MTL-BAM is effective on the JNLPBA dataset on flat entities.

#### 3.3.2. Analysis of Experimental Results of CoNLL2003 Dataset

For the dataset CoNLL2003, the following models will be used for experimental comparison:

The sequence annotation model proposed by Lample et al. [22] uses the BILSTM-CRF model to recognize flat entities. Strubell [23] proposed an iterative dilated convolutional neural network (ID-CNN) for entity recognition, which has better large context and structured prediction capabilities than traditional CNNs. Devlin et al. [24] proposed to use the BERT pretraining model to fine-tune a large amount of pretrained corpus to improve entity recognition. Akbik et al. [25] exploits the internal state of a trained character language model to generate a novel word embedding that enhances contextual representation to improve entity recognition.

The comparison results between the data set CoNLL2003 and the BAM model based on the same experimental environment are shown in Table 9.

On the CoNLL2003 dataset, the *F*1 value of the model in this paper has increased by 0.2%, the recall rate has increased by 0.66%, and the accuracy has decreased to a certain extent. The experimental results verify that the model is equally effective on the flat entity CoNLL2003 and nested entities.

Table 10 shows the experimental comparison results between the MTL-BAM model and the other entity recognition models mentioned above. Moreover, only the F1 value was compared to the CoNLL2003 dataset.

It can be seen from the table that the MTL-BAM model is 0.3% higher than the other entity recognition models. The model proposed in this chapter also has a certain effect on the two flat datasets, which shows that the named entity recognition model based on multi-task learning and double affine mechanism in this chapter is versatile in various datasets. However, for the named entity recognition method of the CoNLL2003 dataset, the MTL-BAM model still has a certain gap compared with the current SOTA method [26], mainly because the method proposed in this paper is more for nested named entities.

## 4. Conclusions

From the various experimental results, it can be found that the use of the multi-task learning framework improves the final performance to a certain extent, whether on the baseline model or on other models studied in the past, and it also proves that the method is effective on the flat entity recognition task. With no performance penalty, it is a general framework that can be used for both nested and flat entity recognition tasks.

In addition, the model in this paper still has many shortcomings. The current use of multi-task learning to build a boundary detection model can positively promote the entity classification module. However, for nested entities, the outer boundary can supervise the boundary of the inner entity, and the information of the inner entity has not yet guided the recognition of the outer entity, resulting in only a small improvement in the entire model. Secondly, this paper only studies flat entities and nested entities, and there are more complex entity types in practical applications, such as discontinuous entities. Therefore, in the next step, we can improve the two-way interaction ability of the two tasks. And study the use of this model or the improved model to solve the recognition of various complex entities and improve the effect of various types of entity recognition.

## Figures and Tables

**Table 1:** MTL-BAM algorithm flow.

Algorithm:	MTL-BAM	
Input:	Original dataset	
Output:	Named entity recognition model	
1:	While (not traversing all original dataset sentences) do	
2:	*X*_*t*_^*lm*^ is obtained using the method followed by BERT	
3:	*X*_*t*_^char^ is obtained using CharCNN network encoding	
4:	The corresponding word vector *X*_*t*_^word^ is obtained through FastText	
5:	Connect *X*_*t*_^*lm*^, *X*_*t*_^char^, *X*_*t*_^word^ to get *X*_*t*_	
6:	While (not traversed all *X*_*t*_) do	
7:	Input BiLSTM layer to get output *h*_*t*_	
8:	Use two multilayer perceptrons for *h*_*t*_ to get *s*(*t*), *e*(*t*)	
9:	While (NER model parameters did not converge) do	
10:	While (not traversed all *s*(*t*), *e*(*t*)) do	
11:	Input biaffine network training to get loss_*b*_	
12:	Input boundary detection module training to get loss_*d*_	
13:	Multi_Loss=loss_*b*_+*α*loss_*d*_	

**Table 2:** Experimental parameter setting.

Parameter	Value
BiLSTM size	200
BiLSTM layer	3
BiLSTM dropout	0.4
MLP size	150
MLP dropout	0.2
BERT size	1024
FastText embedding size	300
CharCNN filter widths	[3–5]
Char embedding size	50
Embeddings dropout	0.5
Optimiser	Adam
Learning rate	0.001

**Table 3:** Experimental results of entity types in GENIA dataset.

Entity type	BAM			MTL-BAM		
	*P* (%)	*R* (%)	*F*1 (%)	*P* (%)	*R* (%)	*F*1 (%)
DNA	**78.80**	75.67	**77.20**	78.35	**75.99**	77.15
RNA	86.79	84.40	85.58	**86.92**	**85.32**	**86.11**
Protein	**82.84**	83.56	83.20	81.85	**85.28**	**83.53**
cell_line	81.08	67.42	73.62	**83.29**	**68.31**	**75.06**
cell_type	**77.02**	74.09	75.50	75.87	**75.25**	**75.56**
Total	**81.25**	79.42	80.33	80.62	**80.68**	**80.65**

**Table 4:** Experimental results of entity types in ACE2004 dataset.

Entity type	BAM			MTL-BAM		
	*P* (%)	*R* (%)	*F*1 (%)	*P* (%)	*R* (%)	*F*1 (%)
LOC	65.69	63.81	64.73	**66.67**	**68.57**	**67.61**
WEA	71.43	46.88	56.60	**85.00**	**53.12**	**65.38**
GPE	83.70	83.94	83.82	**85.79**	**85.20**	**85.49**
PER	**90.82**	89.12	**89.96**	88.35	**91.12**	89.71
FAC	72.63	**62.16**	**66.99**	69.00	**62.16**	65.40
ORG	**83.13**	78.80	80.84	80.39	**81.70**	**81.04**
VEH	88.24	88.24	88.24	**88.89**	**94.12**	**91.43**
Overall	**86.37**	83.70	85.00	84.88	**85.78**	**85.33**

**Table 5:** Experimental results of entity types in ACE2005 dataset.

Entity type	BAM			MTL-BAM		
	*P* (%)	*R* (%)	*F*1 (%)	*P* (%)	*R* (%)	*F*1 (%)
LOC	**65.23**	59.34	62.14	64.71	**61.11**	**62.86**
WEA	**83.18**	85.15	**85.11**	82.69	**86.00**	84.31
GPE	**85.28**	83.15	84.20	84.14	**84.53**	**84.33**
PER	**88.76**	87.74	88.25	86.10	**90.02**	**88.96**
FAC	69.50	72.06	70.76	**73.13**	**72.06**	**72.59**
ORG	**85.27**	76.82	**80.83**	84.04	**77.76**	80.78
VEH	75.64	69.75	72.58	**76.34**	**70.30**	**73.20**
Overall	**84.24**	85.36	84.79	84.23	**86.15**	**85.18**

**Table 6:** Comparison results between MTL-BAM model and other entity recognition models on three nested datasets.

Model	GENIA			ACE2004			ACE2005		
	*P* (%)	*R* (%)	*F*1 (%)	*P* (%)	*R* (%)	*F*1 (%)	*P* (%)	*R* (%)	*F*1 (%)
Katuyar and Cardie [2]	79.8	68.2	73.6	73.6	71.8	72.7	70.6	70.4	70.5
Ju et al. [1]	78.5	71.3	74.7	—	—	—	74.2	70.3	72.2
Zheng et al. [18]	74.5	75.6	75.0	—	—	—	—	—	—
Wang and Lu [4]	77.0	73.3	75.1	78.0	72.4	75.1	76.8	72.3	74.5
Yi et al. [5]	—	—	76.2	—	—	84.7	—	—	82.9
Sohrab and miwa [6]	93.2	64.0	77.1	—	—	—	—	—	—
Jana et al. [19]	—	—	78.3	—	—	84.4	—	—	84.3
MTL-BAM	80.62	80.68	**80.65**	84.88	85.78	**85.33**	84.23	86.15	**85.18**

**Table 7:** Comparison results of MTL-BAM and BAM models on JNLPBA dataset.

Model	*P* (%)	*R* (%)	*F*1 (%)
BAM	**72.82**	79.12	75.84
MTL-BAM	72.42	**80.37**	**76.19**

**Table 8:** Comparison results between MTL-BAM model and other entity recognition models on JNLPBA dataset.

Model	*F*1 (%)
Ju et al. [1]	70.1
Zheng et al. [18]	73.6
Wang et al. [20]	73.52
Song et al. [21]	75.04
MTL-BAM	**76.19**

**Table 9:** Comparison results of MTL-BAM and BAM models on CoNLL2003 dataset.

Model	*P* (%)	*R* (%)	*F*1 (%)
BAM	**92.97**	93.41	93.19
MTL-BAM	92.72	**94.07**	**93.39**

**Table 10:** Comparison results between MTL-BAM model and other entity recognition models on CoNLL2003 dataset.

Model	*F*1 (%)
Lample et al. [22]	90.94
Strubell et al. [23]	90.7
Devlin et al. [24]	92.8
Akbik et al. [25]	93.09
MTL-BAM	**93.39**

## Data Availability

The GENIA dataset is provided by the GENIA website (https://www.geniaproject.org/genia-corpus). The ACE2004 dataset is provided by the LDC website (https://catalog.ldc.upenn.edu/LDC2005T09). The ACE2005 dataset is provided by the LDC website (https://catalog.ldc.upenn.edu/LDC2006T06). The CoNll2003 dataset is provided by the Github website (https://github.com/synalp/NER/tree/master/corpus/CoNLL-2003). The JNLPBA dataset is provided by the Metatext website (https://metatext.io/datasets/jnlpba).
